# Your Physical Activity Is in Your Hand—Objective Activity Tracking Among University Students in Hungary, One of the Most Obese Countries in Europe

**DOI:** 10.3389/fpubh.2021.661471

**Published:** 2021-09-16

**Authors:** Gergely Ráthonyi, Viktor Takács, Róbert Szilágyi, Éva Bácsné Bába, Anetta Müller, Zoltán Bács, Mónika Harangi-Rákos, László Balogh, Kinga Ráthonyi-Odor

**Affiliations:** ^1^Institute of Applied Informatics and Logistics, University of Debrecen, Debrecen, Hungary; ^2^Institute of Sport Management, University of Debrecen, Debrecen, Hungary; ^3^Institute of Accounting and Finance, University of Debrecen, Debrecen, Hungary; ^4^Institute of Rural Development, Regional Economy and Tourism Management, University of Debrecen, Debrecen, Hungary; ^5^Institute of Sport Science Coordination, University of Debrecen, Debrecen, Hungary

**Keywords:** activity trackers, free-living conditions, university students, physical activity, step count

## Abstract

Inadequate physical activity is currently one of the leading risk factors for mortality worldwide. University students are a high-risk group in terms of rates of obesity and lack of physical activity. In recent years, activity trackers have become increasingly popular for measuring physical activity. The aim of the present study is to examine whether university students in Hungary meet the health recommendations (10,000 steps/day) for physical activity and investigate the impact of different variables (semester-exam period, days-weekdays, days, months, sex) on the level of physical activity in free-living conditions for 3 months period. In free-living conditions, 57 healthy university students (male: 25 female: 32 mean age: 19.50 SD = 1.58) wore MiBand 1S activity tracker for 3 months. Independent sample *t*-tests were used to explore differences between sexes. A One-way analysis of variance (ANOVA) was used to explore differences in measures among different grouping variables and step count. A Two-way ANOVA was conducted to test for differences in the number of steps by days of the week, months, seasons and for sex differences. Tukey HSD *post-hoc* tests were used to examine significant differences. Students in the study achieved 10,000 steps per day on 17% of days (minimum: 0%; maximum: 76.5%; median: 11.1%). Unfortunately, 70% of the participants did not comply the 10,000 steps at least 80% of the days studied. No statistical difference were found between sexes. However, significant differences were found between BMI categories (underweight <18.50 kg/m^2^; normal range 18.50–24.99 kg/m^2^; overweight: 25.00–29.99 kg/m^2^ obese > 30 kg/m^2^, the number of steps in the overweight category was significantly lower (*F* = 72.073, *p* < 0.001). The average daily steps were significantly higher in autumn (*t* = 11.457, *p* < 0.001) than in winter. During exam period average steps/day were significantly lower than during fall semester (*t* = 13.696, *p* < 0.001). On weekdays, steps were significantly higher than on weekends (*F* = 14.017, *p* < 0.001), and even within this, the greatest physical activity can be done by the middle of the week. Our data suggest that university students may be priority groups for future physical activity interventions. Commercial activity trackers provide huge amount of data for relatively low cost therefore it has the potential to objectively analyze physical activity and plan interventions.

## Introduction

People's lifestyles have changed significantly in recent years, the number of obese people has increased, mainly in developed countries and Hungary has one of the highest obesity rate in Europe. The Hungarian Central Statistical Office reported that 55.5% of Hungarians aged 15 years and older are obese or overweight (1). A worrying trend is that more and more children and young people are experiencing obesity (2, 3), and according to research, 60% of them will struggle with excess weight by adulthood (4). In recent decades, several research groups have analyzed the relationship between physical activity and health, as a result of which it can be stated today that regular physical activity has a positive effect on the human body (5–8).

Insufficient physical activity is a key risk factor for non-communicable diseases (NCDs) (such as cardiovascular diseases, type 2 diabetes, some cancers, mental health diseases, and chronic respiratory diseases) and is currently one of the leading risk factors for mortality worldwide (9–15). The World Health Organization recommends 150 min of moderate intensity or at least 75 min of vigorous intensity aerobic physical activity, or an equivalent combination of moderate- and vigorous intensity activity throughout the week, for substantial health benefit (16). However, 23% of adults and 81% of adolescents do not achieve the recommended physical activity targets worldwide (16). According to the WHO in Hungary the estimated prevalence of sufficient physical activity levels are 42% among children and adolescence and 31% among adults (17). From childhood to adolescence through adulthood, physical activity steadily decreases (18, 19), therefore university students should be considered a high-risk group. Recent studies have revealed that the prevalence of obesity and cardiovascular diseases among young age groups such as university students have increased, therefore focus on reaching optimal levels of physical activity is very important (20–23). Unfortunately, a high proportion of young people are characterized by sedentary behavior, particularly our target group, who spend most of their time studying, attending lectures, using computers, watching television, or traveling. Immediately after adolescence, university students undergo emotional, physiological and environmental changes influencing aspects of their consumer habits and lifestyle, including their physical activity (24–26). Previous studies have shown that university students are less active and do not meet the WHO recommendations for physical activity levels (26–28). In university students, sitting time can exceed 8–9 h a day (29). It is known that individual factors such as age, sex and health status affect individual's physical activity (30). The characteristics of university life (study/exam periods, walking between buildings) as well as the time and weather conditions (different seasons, weekdays, and weekends) can also affect the physical activity level of students (25, 31–36).

However, many people worldwide do not know whether or not they comply with the above-mentioned WHO recommendation (11). Earlier studies have argued that the level of physical activity is often overestimated by most people, as well as by university students (37, 38). Activity trackers and mobile applications could be potential solutions for this issue, because these devices have the ability to monitor different health behaviors and indicators, for example, physical activity, sedentary behavior, heart rate, blood pressure, and sleep. These devices are able to objectively track physical activity in real time by collecting data through integrated sensors (e.g., accelerometers, gyroscopes) (39–45) and allow users to self-monitor their physical activity against public health recommendations or their own goals (46–48). These devices are small and user friendly, and can measure the number of steps taken and also convert these measurements using algorithms into other measures such as calories burned and distance (49). Activity trackers can contribute to the development of personalized physical activity interventions, and can also help sport scientists and health-related practitioners by providing deep insights into an individual's health and fitness (50–56).

Due to the rapid technological development and the growing awareness of the need to stay healthy and fit, public adoption of wearable activity tracker devices has been increasing over recent years (56, 57). According to recent market reports, the global fitness tracker market was valued at 30 billion USD in 2019 and is forecast to reach 92 billion USD by 2027 (58).

There has been great interest in the use of wearable activity trackers among the scientific research community in recent years. A review study identified six key topics: technology (accuracy, validity, data collection, and analysis), patient treatment and medical settings (monitoring and rehabilitation patients), behavior change (effect on physical activity, or other health-related behaviors), acceptance and adoption (understanding users' rationale behind retaining or abandoning their devices), self-monitoring data (affordances for collection and analysis of personal data), and privacy (increasing concern about how providers of these devices use the data they record, and the personal privacy protections that are afforded to users) (59).

The basic mechanism of the step-counting function is that the acceleration values on 3 orthogonal axes are obtained, from which the secondary wave peaks are monitored after the waves have been filtered, and the number of peaks is the number of steps (60, 61). Therefore, activity trackers provide more detailed information about physical activity than traditional pedometers (61).

Activity trackers differ as to which component of physical activity they measure, but almost every device can measure the most basic component, the steps count. Tracking steps remains an essential element in promoting physical activity, because it is a simple and easy-to-understand method for objective self-monitoring of an individual's level of activity (62). Activity trackers and step counts can be used to infer patterns and levels of physical activity and can also have a significant effect on promoting physical activity (41, 48, 54). Many governing bodies (like the American Heart Association and American College of Sports Medicine) and public health initiatives (like Queensland State Health Department—Queensland Health) suggest taking at least 10,000 steps a day (a commonly used physical activity guideline) for maintaining physical fitness and health (62–64). The Centers for Disease Control states that adults can achieve the moderate level of activity by taking at least 10,000 steps a day (32, 65).

The aim of the present study is to examine whether university students in Hungary meet the physical activity guideline of 10,000 steps per day, and investigate the impact of different variables (semester-exam period, days-weekdays, days, months, sex) on the level of physical activity in free-living conditions for 3 months period.

## Methods

### Overview

This present study has gathered data from wearable activity trackers among university students. A questionnaire survey was performed in order to collect demographic data and basic information about the participants. Experiments were carried out from November 2018 to January 2019. The study protocol was approved by the Regional Ethics Board (code: DE RKEB/IKEB: 5187-2019) at the Clinical Center of the University of Debrecen (Hungary). All participants provided informed consent in compliance with the principles of the Declaration of Helsinki and the General Data Protection Regulation (GDPR).

### Study Sample and Procedure

Only healthy volunteer university students were included in the study. They were recruited via different courses at the University of Debrecen where they were informed about the exact process of the investigation. There were several inclusion criteria: >18 years of age, no critical illnesses, not allergic to rubber, and willingness to continuously wear the device. The subjects were required to wear the device and maintain normal living conditions for three consecutive months (a minimum of 80 days) including weekends. During the 92 days the normal living conditions of the university students (walking, climbing stairs, sitting, traveling, doing sport etc.) could be accurately represented.

Participants attended an appointment at which a survey was conducted and the MiBand 1S bracelets were provided. Beyond observing demographic data, the questionnaire was designed to explore the students' sports habits and perceived health status. Their body mass index (kg/m^2^) was also calculated from weight and height values; the index is commonly used to classify categories in adults in the following way: Underweight <18.50 kg/m^2^; Normal range 18.50–24.99 kg/m^2^; Overweight: 25.00–29.99 kg/m^2^; Obese > 30 kg/m^2^ (66).

MiBand 1S activity trackers were provided on loan for the semester, to each student for the duration of the study. At the end of the study participants had to return the trackers. Bracelets were worn on the non-dominant wrist to ensure a stable position and settings, in order to maintain consistency. Participants were asked to synchronize data between wearable devices and the mobile app at least once a day, and to send the step count data—which could be exported from a third party mobile application—every second week. The MiBand 1S activity tracker has some important attributes, which distinguish it from other devices in the market: it can operate for up to 20–30 days on a single charge (67), and the raw data can be easily extracted, therefore researchers can carry out deeper data analysis (68). We have chosen MiBand 1S for our study because it is a cheap, reliable and accurate activity tracker in the measurement of steps (39, 50, 52, 65, 69–71).

Sixty-three students were recruited in the study and researchers excluded data from 2 subjects due to device failure, which resulted in a sample of 61 students. After applying the a priori data inclusion criteria (>80 days), 57 students remained in the sample. In order to minimize the attrition rate only those students were recruited in the study, who had the willingness to continuously wear the device. The project team kept in touch with the students on every second week in order to remind them to synchronize the application and the activity tracker and helped them with any technical problem. In the database more than 500,000 data points were entered.

### Physical Activity Categories

According to measured daily step count, the following physical activity categories have been distinguished by Tudor Locke (Table 1). Tudor-Locke and Bassett (63) originally proposed that <5,000 steps/day is a sedentary behavior, but recognizing the floor effect they suggested that the original sedentary level could be further divided into two additional categories.

**Table 1:** Physical activity categories according to step count.

**Category**	**Step count**
Basally active	<2,500
Limited active	2,500–4,999
Low active	5,000–7,499
Somewhat active	7,500–9,999
Active	10,000–12,499
Highly active	>12,500

*Source: (51, 52)*.

This step index does not take into consideration age or chronic disease/disability, but can be used as an absolute classification and is adequate for the purposes of this present study (62).

### Data Management

Different mobile operation systems and different mobile applications generate different types of data related to step counts. Raw step data came from the participants via emails, which contains the step data from the activity trackers and are extracted with phone apps measured at 10 min intervals, but they are different both semantically and in terms of granularity. The step data extracted with Android phone app are running totals by days, and measured at different times. The step data from iOS sources are the steps made at the given standardized 10 min intervals (0:00, 0:10, 0:20, …, 23:50) and step data are only available for intervals when there was motion. In order to create a comparable and analyzable database we should standardize the different types of data; therefore Online Analytical Processing (OLAP) methodology and a hybrid design methodology with formal descriptive techniques have been used.

A detailed presentation of the following data management methodology with the detailed formalized description can be found in Takács et al. (72).

In the present study the authors used the Visualized Management Question-based Design methodology to creating the database; this methodology is very similar to the Goal/Question/Metric-based Methodology (referred to as GQM), which is a proven method for driving goal-oriented measures. With GQM, we start by defining the goals we are trying to achieve, then clarifying the questions we are trying to answer with the data we collect. Although there are some differences. The process included the following steps.

- Requirement analysis
- Minimal granularity
- Ideal schema
- Source analysis
- Integration
- Dimensionality integration
- Multidimensional modeling

In the requirement analysis phase, we defined the research questions with metrics and dimensionality of the problem also with the required visualizations. Then we formalize these specifications with our special structured stenography that is based on the terminology corresponding to the current problem. The output of this step is a set of formalized questions. Based on the set of formalized questions we specify the required minimal granularity for every indicator. The output of this step is the set of indicators with minimally detailed dimensions.

After that we map the dimensional attributes and values to keys, produce the initial conceptual schemata. The output of this step contains ideal dimensions (keys, attributes and hierarchies) and ideal facts (with dimension keys for join), independently from the sources. The main question of the source analysis step is: What kind of transactions can we get them from? We decompose ideal facts into potential elementary transactional attributes and identify them in the source systems. The output of this step is the derived potential schemata. The ideal schemata from the requirement analysis are compared with potential star schemata. Match occurs, when the two schemata contain the same fact, and, both have the same dimensionality in the same granularity level. In this step we define required transformations and calculate fact tables and common dimensions with attributes.

Finally at the multidimensional modeling we build the cube(s) with dimensions, dimension hierarchies and measures (72).

### Statistical Analysis

Statistical analysis was performed using SPSS software (version 21.0, Chicago, Illinois, USA) and at all statistical tests, statistical significance was defined as 5%. First, a box plot and Kolmogorov-Smirnov test were conducted for each measurement indicator to examine the distribution. Descriptive statistics were calculated for the demographic variables. Pearson chi-square analysis was used to examine for significant differences in the proportion of physical activity recommendation criteria and sex.

Independent sample t-tests were used to explore differences between sexes. A One-way analysis of variance (ANOVA) was used to explore differences in measures among different grouping variables and step count. A Two-way ANOVA was conducted to test for differences in the number of steps by days of the week, months, seasons, and for sex differences. Tukey HSD *post-hoc* tests were used to examine significant differences (73).

## Results

During the fall 2019 semester 57 full-time undergraduate students (min age = 18, max age = 25, mean age = 19.54, SD = 1.43) finally participated in this study. There were no significant differences in age between the 25 male and 32 female students (*t* = 1.154, *p* = 0.568). In connection with the perceived health status there is a significant difference between the sexes (*t* = 2.737, *p* = 0.006). Male students evaluate their health status higher than female students (Table 2).

**Table 2:** Characteristics of the voluntary university students.

**Category**	**Total group (SD)**	**Male (SD)**	**Female (SD)**	***p*-value**
Age (average)	19.50 (1.58)	19.53 (1.43)	19.48 (1.68)	0.568
Perceived health status	3.99 (0.58)	4.02 (0.43)	3.97 (0.68)	0.006
BMI	22.48 (3.17)	22.72 (3.15)	22.29 (3.18)	0.000
**Category**	**Total group %**	**Male %**	**Female %**	***p*** **-value**
Number of days–meet physical activity recommendation criteria (10,000 steps/day)	17.1%	16.3%	17.8%	0.236

As far as BMI value is concerned (*t* = 3.977, *p* < 0.001) male students have a significantly higher value when compared to female students. Seventy-five percent of the participants have normal weight and only one third of them are overweight (19.5%) or underweight (5.5%).

In terms of step count, we first examined how students perform in terms of completing 10,000 steps per day. Students in the study achieved 10,000 steps per day on 17% of days (minimum: 0%; maximum: 76.5%; median: 11.1%). Unfortunately, 70% of the participants did not complete the 10,000 steps on at least 80% of the studied days. In the examined period nobody completed the daily recommendation and only one student took 10,000 steps/day on seven consecutive days in the 3 month period. However, when the frequency requirement was decreased to 5 days/week, only 5 students achieved this target at least once during the 3 months. In November 16% (9 students), in December 7% (4 students) and in January only 3.5% (2 students) of the students reach the average 10,000 steps/day. From November to December the average number of monthly steps decreased by 76% of the students. The average rate of this reduction was 25%. On the other hand in this period 24% of the students were able to increase their average monthly step counts with 17%. From December to January the average number of monthly steps decreased by 74% of the students. The average rate of this reduction was 26%. On the other hand in this period 26% of the students were able to increase their average monthly step counts. From November to January the average number of monthly steps decreased by 87% of the students. The average rate of this reduction was 38%.

As Table 3 shows, we found no significant difference in the average daily number of steps by sex (*t* = 1.424, *p* = 0.155). The interaction between sex and the other variables was not detected; therefore the analyses of other grouping variables were performed for males and females together. In terms of BMI categories, we experienced signficant differences. Students in the normal weight category have the highest step count per day, while the number of steps in the overweight category was significantly lower (*F* = 72.073, *p* < 0.001). *Post-hoc* analysis revealed that there was no significant difference between the normal and underweight categories (*p* = 0.085). If we look at the data by season, the average daily number of steps was significantly higher in autumn (*t* = 11.457, *p* < 0.001) than in winter, which is not surprising. With the onset of cooler months, we can see a steady decline in physical activity. The examination session can also be included in this period, during which the step data were also lower than during the fall semester (*t* = 13.696, *p* < 0.001). On weekdays, data were significantly higher than on weekends, and even within this, the greatest physical activity can be observed in the middle of the week. As a result of the analysis of variance and *post-hoc* tests, it can be stated that there is a significant difference between the average number of steps on weekdays and weekends (*F* = 14.017, *p* < 0.001); however, there was no justifiable difference between weekdays (from Monday to Friday) and weekends (Saturday and Sunday). On weekend days significant difference (*t* = 3,365, *p* < 0.001) was observed between sexes, male students (5,914 steps) had more average steps on weekend days compared to female (4,904 steps) students. On weekdays, no significant difference was found regards to the average number of steps between the sexes (male = 6,765; female = 7,034).

**Table 3:** Steps/day differences among diverse categories.

**Category**	**Step count (SD)**	**p-value**
**Total group**	6,486.06 (5,234.16)	
**Sex**		0.655
Male	6,529.60 (4,490.91)	
Female	6,449.44 (5,786.83)	
**BMI categories**		0.000
Underweight	6,200.39 (4,857.31)	
Normal weight	6,776.12 (5,220.32)	
Obese	4,623.91 (3,472.24)	
**Season**		0.000
Autumn	8,033.91 (5,261.91)	
Winter	5,830.95 (5,083.39)	
**Month**		0.000
November	7,873.68 (5,046.81)	
December	5,928.31 (4,508.38)	
January	5,435.81 (5,169.39)	
**Day**		0.000
Monday	6,391.22 (4,974.46)	
Tuesday	6,778.82 (5,049.37)	
Wednesday	7,235.73 (5,115.96)	
Thursday	7,073.58 (5,413.95)	
Friday	7,118.03 (6,108.03)	
Saturday	5,372.62 (5,017.16)	
Sunday	5,203.83 (4,389.3)	
**Weekday/Weekend**		0.000
Weekdays	6,921.21 (5,346.24)	
Weekends	5,288.22 (4,711.87)	
**Educational period**		0.000
Study period	7,715.07 (5,404.85)	
Exam period	5,214.77 (4,724.98)	
**Sport**		0.000
Yes	6,749.88 (5,391.81)	
No	5,326.57 (4,278.97)	

## Discussion

To the best of our knowledge, there have been few studies analyzing objective physical activity in university students (25–27, 32, 74–76) and even fewer involving a consumer based activity tracker over longer periods. Studying this specific population is particularly interesting because university students are a high-risk group for obesity and low levels of physical activity as a result of their change of behavior and lifestyle after leaving the well-controlled environment of the secondary school (24).

The present study reveals that most of the time university students do not fulfill the compliance to get the daily average 10,000 steps. In our study the average steps/day of the total population is 6,486 on weekdays 6,921 and on weekend days 5,288, which are very low and an alarming result in contrast to those reported in other studies (25, 26, 75, 76). According to Tudor-Locke and Bassett physical activity categories this average step count is in the low activity level (63). In contrast to our results Clement et al. (26) examination have shown that Portuguese university students have an average 10,011 steps/day on weekdays and 6,622 steps/day on weekend days. Arias-Palencia et al. (25) examined Spanish university students and found that they have an average 9,081 steps/day on weekdays and 7,971 steps/day on weekend days. Our low daily average step count predicted the result that nobody completed the daily recommendation, and only one student took 10,000 steps/day on seven consecutive days in the 3 month period. However, when the frequency requirement was decreased to 5 days/week, 5 students achieved this target.

Although previous studies found that male students took statistically more steps than female students, in the present study no statistical differences were found between the sexes when examining the total average (77, 78). Nevertheless, examining daily average steps in more detail, significant differences were found between the sexes only on the weekend days: on weekend days, male students walked statistically more steps. Similar to other researches (25, 33) on weekdays no significant differences were found; university students probably have similar activity habits due to the stable timetable set by their academic classes.

It is well-documented that the season and poor weather conditions can have an impact on the level of physical activity. Previous research has found that weather conditions and season may be a barrier to physical activity (32, 34, 35). For example in the summer time people may take more steps. The present study revealed that in the autumn (November) participants had more average steps per day than in the winter (December, January). At this time of year (measurement) temperatures fall steadily and the amount of precipitation increases. A decline can be observed in step counts from autumn to winter, which could be due to less walking across campus, or the fewer opportunities for leisure-time activities, or the end of the study period. In December holidays could also contribute the lower level of daily steps.

A significant decrease was observed in step count from weekdays to weekend days. Similar to other studies university students were more active on weekdays than weekends (25, 31, 33). Possible reasons of the higher rates of the weekday step counts could be that students have to walk across the campus to their academic classes, or the compulsory physical education lectures.

A significant decrease was observed in step count from study to exam periods. At the end of the study period, the walking time across the campus is drastically diminished, the university campus noticeably have become empty and students only visit the campus on the occasion of exams. This could be the main reason for detecting fewer steps in the exam period. On the other hand the characteristics of the season and the weather conditions could also contribute to the lower level of physical activity (32, 34). The findings of this study reveal a statistically significant decrease in the exam period in steps/day in both sexes on weekdays and weekend days as well.

The results of the present study may have important practical applications for exercise specialists and other professionals dealing with physical activity programs and interventions in universities. University experts should concentrate on increasing physical activity by providing opportunities for students to be active and participate in various free physical activity programs, particularly on weekends, during the exam period and the winter months, when the level of physical activity decreases. The time spent at university is a very important life stage in students' lives in terms of physical activity, because this is the last chance to provide quality physical education and develop behavior patterns in order to keep them active throughout their life. This is very important because recent studies have revealed that the prevalence of obesity and cardiovascular diseases among young age groups such as university students have increased (17–20).

### Limitation

However, the present study has some limitations, which must be taken into consideration when interpreting the results. On the one hand, this study focused on a small element of physical activity and worked with a small sample of students, which could cause bias. In the present study only 57 participants were analyzed; therefore, any generalization of our results should be treated with caution. On the other hand, another shortcoming could be the youth of the sample group. We can assume that this population has higher physical activity levels, and therefore the results should not be generalized to all age groups. It also emerges from the characteristics of this study that the season of the data collection (fall, winter) may be an issue; therefore, it is possible that the number of step counts may be different in another season (like in summer or spring), as occurred in previous studies presented above. As far as activity tracker devices are concerned, the enormous amount of collected data has been limited by several challenges, which can also be a limitation for the present study as well. The MiBand 1S device has certain limitations, such as limited waterproofing and how it can be worn (as a bracelet). Due to the variety of devices and sensors included in fitness activity trackers and the algorithmic procedures, there are many differences related to the accuracy of each device. Measurement and technical problems can sometimes occur with the devices, for example, related to cycling or the walking styles or body dimensions. The devices were on loan only for the study period, and this is likely to have impacted on results compared with other populations, such as those who bought their own devices (79).

### Conclusion

Our results revealed that participants fall behind the popular target of 10,000 steps per day, which help to achieve the WHO physical activity recommendation (16). Students were more active on weekdays than on weekends, more active in study periods than exam periods and more active in autumn than winter. A significant difference was found between the sexes regards to the average steps only on the weekend days, when male students were more active.

An accurate objective level of physical activity is important for university professionals' in order to increase students physical activity, prevent obesity and reduce the risk of future diseases.

## Data Availability Statement

The raw data supporting the conclusions of this article will be made available by the authors, without undue reservation.

## Ethics Statement

The studies involving human participants were reviewed and approved by Regional Ethics Board at the Clinical Center of the University of Debrecen (code: DE RKEB/IKEB: 5187-2019). The patients/participants provided their written informed consent to participate in this study.

## Author Contributions

GR, ZB, ÉBB, KR-O, and MH-R contributed to the design and implementation of the research. GR, VT, and ÉBB managed and analyzed the data. ÉBB, KR-O, RS, GR, VT, MH-R, AM, LB, and ZB contributed to the interpretation of the results. GR, VT, and KR-O wrote the manuscript. KR-O, ÉBB, and GR edited the manuscript. GR, ÉBB, LB, and KR-O made the supervision of the manuscript. All authors provided critical feedback and helped shape the manuscript and read and agreed to the published version of the manuscript.

## Funding

This publication was supported by the GINOP-2.3.2-15-2016-00005 project. The project was co-financed by the European Union under the European Regional Development Fund.

## Conflict of Interest

The authors declare that the research was conducted in the absence of any commercial or financial relationships that could be construed as a potential conflict of interest.

## Publisher's Note

All claims expressed in this article are solely those of the authors and do not necessarily represent those of their affiliated organizations, or those of the publisher, the editors and the reviewers. Any product that may be evaluated in this article, or claim that may be made by its manufacturer, is not guaranteed or endorsed by the publisher.
